# Characterization of ancient DNA preservation in copper-patinated human bone and tooth samples from Latvia

**DOI:** 10.1128/spectrum.02705-24

**Published:** 2025-08-12

**Authors:** Alise Pokšāne, Jānis Ķimsis, Elīna Pētersone-Gordina, Antonija Vilcāne, Guntis Gerhards, Renāte Ranka

**Affiliations:** 1 Latvian Biomedical Research and Study Centre 382968, Riga, Latvia; 2 Institute of Latvian History, University of Latvia 618043, Riga, Latvia; Luonnonvarakeskus, Oulu, Finland

**Keywords:** aDNA, patina, bronze

## Abstract

Bronze and copper-containing alloys are known to possess antibacterial properties, which, besides mineralization, might contribute to the observation of better preservation of bone, teeth, and other organic materials in close proximity to bronze artifacts in archaeological contexts. Often, the selection of preserved hard tissue material from burials for ancient DNA (aDNA) analysis is limited due to the sampling strategies used at the time of excavations or poor preservation in general. This study assessed the suitability of samples with copper patination for in-depth human and bacterial aDNA analysis. To characterize the difference between patinated and unpatinated samples, a qualitative assessment of human DNA preservation and metagenomic analysis using Kraken2 and MEGAN Alignment Tool was performed. For patinated samples, a very low proportion of endogenous DNA preservation was observed. Only one—the least patinated—out of five analyzed samples could be authenticated to be containing human aDNA with confidence. A patinated sample of specific bacterial genera was affiliated with soil and aquatic, mostly marine environmental, microorganisms. In conclusion, due to mineralization processes that occur during the patination of hard tissue, endogenous DNA preservation is seen to be greatly affected, thus making heavily patinated samples a compromised source of endogenous aDNA. Future in-depth research is needed, including the assessment of chemical composition of patinated tissues.

**IMPORTANCE:**

Ancient DNA research has recently become a very powerful tool for archaeological and historical research, enabling the discovery of information about various aspects of our predecessors’ lives, but it is limited by the availability of material to be sampled. To our knowledge, there is no previous study focused on effects of copper patination on ancient DNA preservation and metagenomic profiles of archaeological teeth and bone samples. Our results suggest that patination should be considered an influential factor during sample selection, as it affected human endogenous DNA preservation and metagenomic diversity within analyzed samples.

## INTRODUCTION

Bronze is a copper alloy which has been used by humanity since antiquity for different applications from weaponry to decoration (1, 2). As a result of copper oxidation or copper-containing alloys, a green patina called *verdigris* is formed on the surface of the metal, which historically has been widely used as a pigment because of its distinct color (3). Patina could also be observed on such organic materials as bones or teeth which have been found close to bronze artifacts, and it has been observed that in archaeological contexts, osteological and other organic materials exhibit a tendency to have better visual integrity in close proximity to bronze due to induration (1, 4–6).

Poor bone preservation, which affects the availability of sampling material, is a highly limiting factor for possible genetic analysis of ancient populations or individuals of interest. For some burial sites with poor preservation of bones and teeth, a part of osteological material available with best visual integrity can be found in the presence of bronze (1), but little is known on its effects on the survival of endogenous ancient DNA (aDNA) in these samples. Forensic studies of the preservation and degradation of modern DNA indicated the problematic nature of possible amplification inhibition in cases when sampling material has been affected by copper- and zinc-containing metal alloys (7, 8). However, to our best knowledge, studies on the effect of bronze on the exploitability of patinated osteological material for aDNA analysis have not been reported yet.

Copper and its alloys possess antibacterial properties (9); thus, effects on soil microbial communities colonizing the archaeological samples could be expected, which would further affect the preserved microbiome profiles and even the potential for the extraction of authentic bacterial aDNA.

However, data on the taxonomic diversity of microorganisms colonizing patinated osteological material are scarce. Metagenomic studies of copper-containing metal patina samples of historic origin are limited to modern DNA molecules of microorganisms which are able to survive on antibacterial surfaces and display a small taxonomic diversity if the DNA can be extracted at all (10). As these studies have used only patina *per se* (10) buccal cell suspensions and saliva samples which were intentionally contaminated with copper and other compounds (7, 8), there is no clear evidence if these findings about problems with DNA extraction and PCR inhibition could be extrapolated to archaeological bone and tooth samples.

Overall, there is a paucity of research regarding the use or even usability of problematic and chemically contaminated hard tissue samples such as patinated bones or teeth in aDNA studies. The aim of this study was to examine the effects of copper patina on the endogenous and microbial DNA preservation in archaeologically obtained osteological material.

## MATERIALS AND METHODS

### Sample preparation

Archaeological material was obtained from the Lejasbitēni cemetery (7th to 10th century CE), located on the right bank of the river Daugava (Fig. S1) (11). Archaeological dating of the site, which was mainly based on internal spatial organization of the cemetery and grave goods (11), is supported by previously published C^14^ dating results (12) . The bone and tooth material was excavated during several seasons between 1961 and 1964 under the supervision of Vladislavs Urtāns (11), and in line with the common archaeological practice at the time, mainly skulls were collected. The age of adult individuals was therefore assessed based on skeletal fusion, including cranial suture closure (13), while in non-adults, dental development (14) and eruption (15) were used . All individuals estimated to be under 19–20 years were considered non-adults (13).

**Fig 1:**
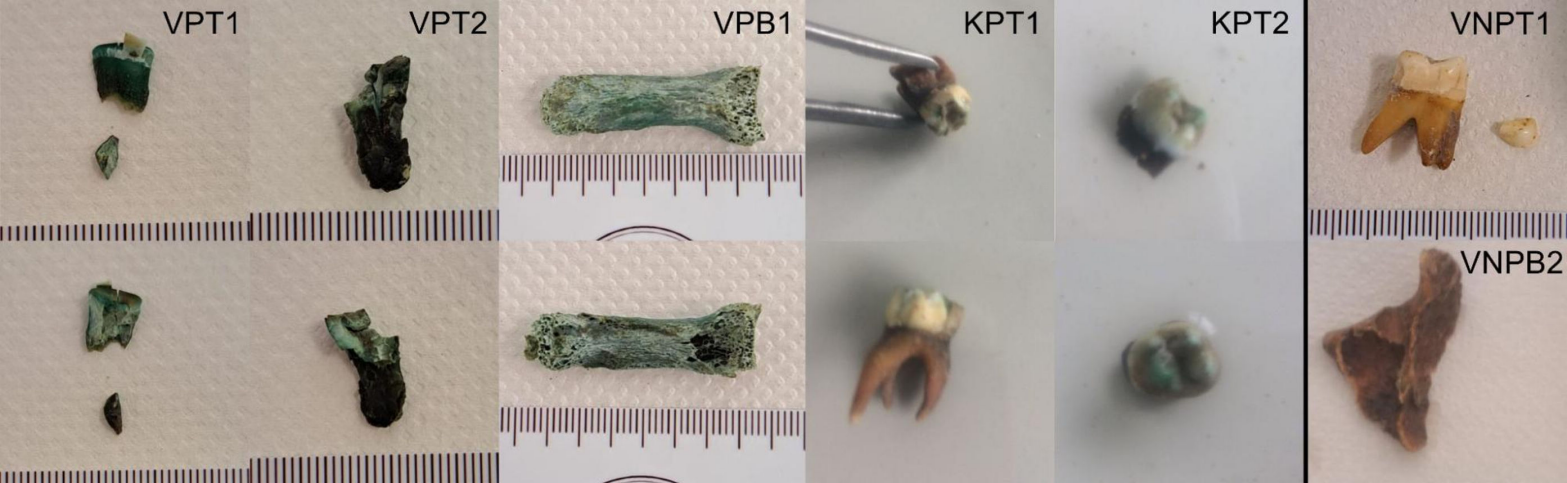
Bone and tooth samples with copper patination used in this study. VPT1 and VPT2 are displayed after the cutting step. Behind the black line, two samples without copper patination are displayed for comparison.

For this study, patinated bones (Fig. 1) and teeth were sampled from five different individuals (three adults and two non-adults), and unpatinated osteological material—from five other individuals (three adults and two non-adults) buried in the same cemetery.

Sample processing took place in special clean room facilities in the Latvian Biomedical research and study center, following aDNA-specific research precautions (16).

Before DNA extraction, all samples were washed with 6% sodium hypochlorite, double distilled water, and 70% ethanol in the same order. After washing, the samples were treated with UV lights for 30 minutes from each side and then left to dry for several days.

The bone powder was collected using a Dremel drill by removing the surface layer of the sampled osteological element and targeting the densest regions of the bone. Tooth samples were cut in half, exposing the pulp. One half of each tooth was incubated in whole for the DNA extraction. The total weight of osteological material for a single extraction was between 30 and 100 mg.

Since the remains found during the excavation were older than 100 years, no permissions were required for their research in accordance with the national law “On the Protection of the Body of Deceased Human Beings and the Use of Human Tissues and Organs in Medicine” (17).

### DNA extraction and library preparation

DNA extraction was carried out following two distinct methods: protocol by Keyser-Tracqui and Ludes (18) (designated here as Protocol K) and slightly modified Velsko et al. (19) protocol which is adaptation of Dabney et al. (20) (designated here as Protocol V). We modified Protocol V by adding a 15-minute preincubation step at 50°C and an additional overnight incubation step with an increased temperature to 55°C to completely dissolve the bone powder (21–23). DNA was extracted from the pooled supernatants except the supernatant of the preincubation step.

DNA libraries were created using the QIAseq Ultralow Input Kit (Qiagen, Cat. No. 180497) following manufacturer’s recommendations. For library amplification, reagents from the QIAseq kit were used, which includes a polymerase unable to amplify DNA fragments which contain uracil.

DNA concentrations were estimated using the Qubit dsDNA HS Assay Kit (Life Technologies) and Agilent 2100 Bioanalyzer with the High Sensitivity DNA Kit (Agilent Technologies) for DNA fragment size distribution assessment.

Adapter dimers were removed using the Nucleomag NGS Clean-Up and Size Select Kit (Macherey-Nagel).

DNA libraries were sequenced on a MiSeq (Illumina) sequencer using a MiSeq v2 300 cycle kits with default parameters. Due to the technical limitations, all Protocol V samples were single-end sequenced. In total, five negative controls were prepared and processed for this study, with three extraction and two library construction blanks.

### Preprocessing of sequences

Demultiplexed sequence data for single-end sequenced data were trimmed using *Trimmomatic* version 0.39 (24) with parameters *ILLUMINACLIP:TruSeq3-SE.fa:2:30:10 SLIDINGWINDOW:4:20 MINLEN:20*.

For paired-end sequencing data, merging and trimming steps were performed using ClipandMerge version 1.7.8 (25) with parameters -f AGATCGGAAGAGCACACGTCTGAACTCCAGTCAC -r AGATCGGAAGAGCGTCGTGTAGGGAAAGAGTGTA -l 25 -qt -q 20.

After trimming, all samples were filtered from reads shorter than 33 bp.

### Human aDNA preservation estimation

Reads were aligned to human genome reference GRCh37 with rCRS mtDNA genome reference using *bwa aln* (with parameters *-l 1024 -n 0.01 -o 2*) (26). The aligned reads were deduplicated using *Picard MarkDuplicates* (27) and their quality filtered with *samtools view* (28) leaving only reads with a mapping quality of at least 30 for further analysis.

Assessment of cytosine deamination patterns was carried out using the *mapDamage2.0* program (29). Additional damage pattern detection was carried out assessing the proportion of human genome aligning reads using PMDtools (30), as an additional approximate contamination estimation step (31).

Metrics of human-aligned reads for each of the analyzed individuals were obtained using the *samtools* program with the command *coverage* (28).

Contamination estimation for mitochondrial reads was attempted using Schmutzi (32) and ContaMix (33). Male X-chromosomal contamination estimation analysis was attempted as well (34, 35).

### Microbiome analysis

Trimmed fastq files were deduplicated using the *seqkit* program *rmdup* command (with parameter *-s*) (36) and trimmed 5 bp from each end to lessen the possibility of inaccurate mapping due to the damage at the ends of reads (37).

For the assessment of metagenomic profiles, the sequenced data were aligned to references from extensive reference databases using two different tools*—MEGAN Alignment Tool* (*MALT*) (38) and *Kraken2* (39).

*MALT* analysis was performed using the parameters *–mode BlastN -id 95 -mq 1 -sp 1 –alignementType Semiglobal*, and the database used for this analysis consisted of 22,843 assemblies, which represent bacterial sequences from the NCBI available on 10 December 2020. Decontamination of analyzed samples was performed using metagenomic alignment data from negative controls analyzed with microDecon (40).

*Kraken2* analysis was performed with default parameters, and standard *Kraken* database was used. The obtained output was reorganized using *Bracken* 2.6.2 (41) for a more precise taxonomic assignment of reads. Decontamination of analyzed samples with *microDecon* was also performed (40).

After the removal of references with most reads aligned from negative controls, *Microbiome Analyst 2.0* was used to visualize the composition of final output data and characterize microbial diversity (42, 43).

## RESULTS

### Characterization of endogenous DNA preservation

On average, 1.4 million reads were generated per Protocol V processed samples (Table S1). For patinated tooth material (VPT2 and VPT1), after deduplication and quality filtering, the final endogenous DNA percentage (0.010% and 0.045%) was lower than for unpatinated teeth (VNPT2 and VNPT1) (1.182% and 2.210%). On the contrary, the final endogenous human DNA proportion in the VPB1 bone sample (0.275%) was slightly higher than for unpatinated bone tissue samples VNPB1 and VNPB2 (0.208% and 0.231%) (Table 1). Among Protocol V tooth samples, VPT1 and VPT2 (0.049% and 0%) displayed much lower human-aligning read duplication ratio than unpatinated VNPT1 and VNPT2 (0.454% and 1.551%). Duplication ratio difference between patinated and unpatinated bone samples was not observed (VPB1, 0.512%; VNPB1, 0.250%; and VNPB2, 0.764%) (Table S1).

**TABLE 1:** Summary of metrics characterizing sequenced DNA samples obtained from archaeological samples and negative controls^*a*^

DNA isolation protocol used	Reference	Sample type	Sampled hard tissue	Estimated age of the individual	Patination intensity	Code	Number (%) of quality-filtered human genome and mtDNA reference aligning deduplicated reads	DNA damage pattern	Proportion (%) of human genome and mtDNA aligning deduplicated reads with PMD score ≥3
Protocol V	Velsko et al., 2020 (19 modified from 20)	Tooth	Tooth	Adult	Severe	VPT1	773 (0.045)	Absent	11 (1.423)
			Tooth	Adult	Severe	VPT2	147 (0.009)	Absent	4 (2.721)
			Tooth	Adult	None	VNPT1	19,967 (2.210)	Present	997 (4.993)
			Tooth	Non-adult	None	VNPT2	17,839 (1.182)	Inconclusive	262 (1.469)
		Bone	Phalanx	Adult	Severe	VPB1	3,827 (0.275)	Inconclusive	54 (1.411)
			Cranial fragment	Adult	None	VNPB1	3,132 (0.208)	Present	484 (15.453)
			Cranial fragment	Adult	None	VNPB2	2,968 (0.231)	Present	300 (10.108)
		Negative control	na^*b*^	na	na	VNCE1	478 (2.997)	Absent	1 (0.209)
			na	na	na	VNCL1	5 (0.780)	Absent	0 (0)
			na	na	na	VNCE2	1,849 (8.652)	Absent	14 (0.757)
			na	na	na	VNCL2	3 (1.277)	Absent	0 (0)
Protocol K	Keyser-Tracqui and Ludes, 2005 (18)	Tooth	Tooth	Non-adult	Partial	KPT1	5,277 (0.019)	Present	303 (5.742)
			Tooth	Non-adult	Severe	KPT2	240 (0.001)	Absent	18 (7.500)
			Tooth	Non-adult	None	KNPT1	8,909 (0.039)	Present	385 (4.321)
		Negative control	na	na	na	KNC1	588 (0.071)	Absent	6 (1.020)

^*a*^ Full table is available in Supplementary material (Table S1).

^*b*^ na, not applicable.

Protocol K samples were sequenced to average 26 million reads (Table S1).

Endogenous human DNA proportions for protocol K tooth samples KPT1 (0.019%) and KPT2 (0.001%) were smaller than for unpatinated KNPT1 (0.039%) (Table 1). Overall, a higher human-aligning read duplication ratio was observed for all three Protocol K samples. The lowest ratio (4.104%) was identified for severely patinated KPT2, while duplication for KPT1 (6.006%) was quite similar to KNPT1 (7.081%%) (Table S1).

**Fig 2:**
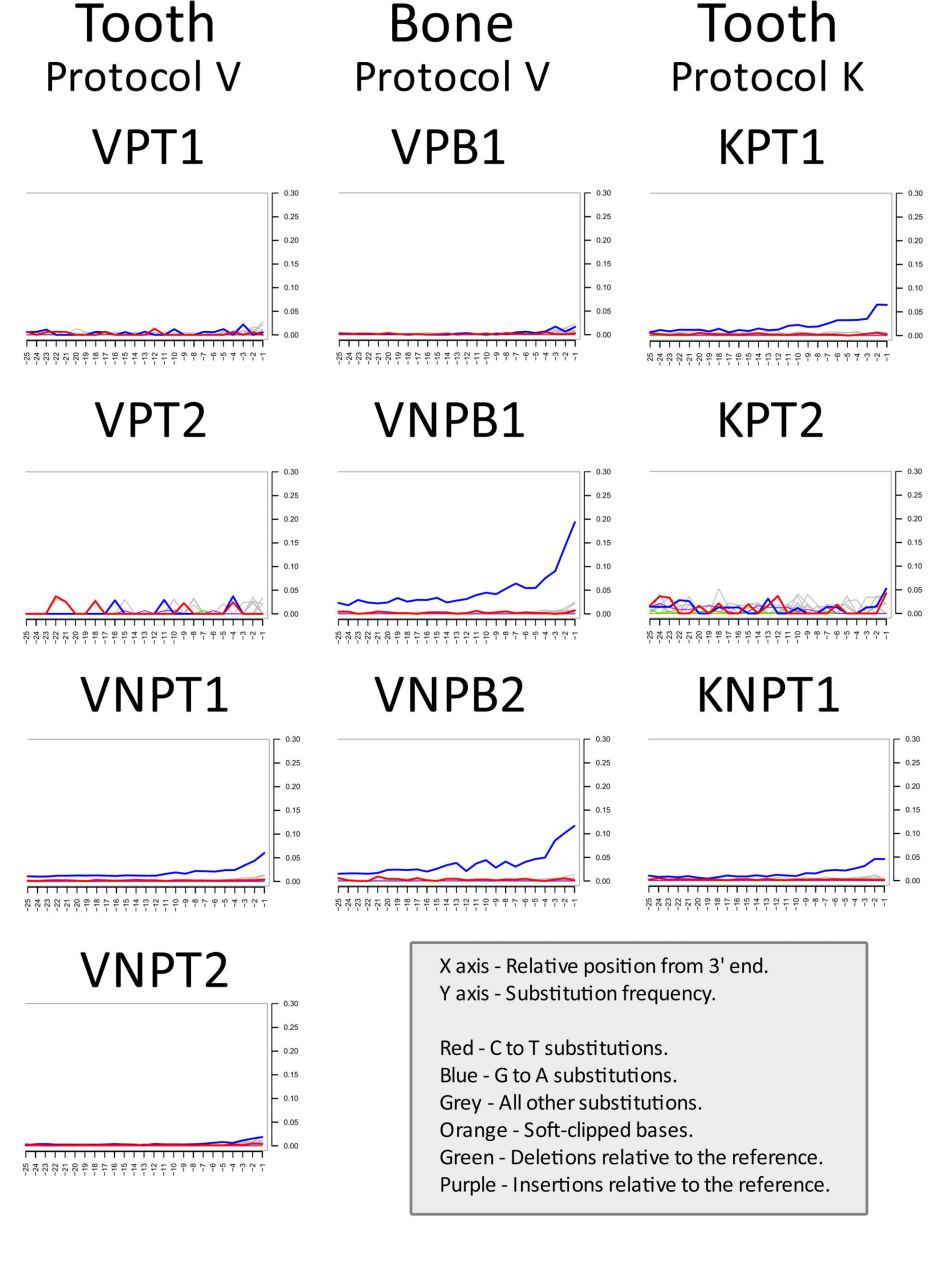
mapDamage plots of reads aligning to the human reference genome. Due to the polymerase included in the library construction kit for this study, damage patterns could be observed only at the 3’ ends, which are displayed in the panel. Full profiles of all processed libraries are displayed in Fig. S2 to S5.

From the patinated samples, only one (KPT1) displayed convincing aDNA-characteristic cytosine deamination at the 3′ end of DNA fragments, while the four others (VPT1, VPT2, VPB1, and KPT2), regardless of the DNA isolation protocol used, did not show a convincing damage pattern, indicating either a false positive alignment of reads or possible modern DNA contamination (Fig. 2; Fig. S3 to S5).

Contrary to the patinated bone and tooth samples, four of five unpatinated samples did exhibit authentic aDNA damage patterns, indicating a possible alignment of authentic ancient human reads (Fig. 2; Fig. S3 to S5).

None of the more common mitochondrial contamination estimation analysis tools (Schmutzi and ContamMix) and male X-chromosomal contamination estimation tool were suitable to properly process our alignment data (Table S1); therefore, we could not assess contamination rates directly.

When tested for aDNA pattern presence in individual sequencing reads, the proportion of reads with postmortem degradation (PMD) score ≥3 varied between the sample groups. For Protocol V tooth samples, the proportion of damaged reads in patinated samples VPT1 and VPT2 (1.423% and 2.721%) was lower than for unpatinated VNPT1 (4.993%), but similar to VNPT2 (1.469%). Protocol V patinated bone tissue sample VPB1, when compared to unpatinated VNPB1 and VNPB2 samples, displayed a substantial decrease in damaged read proportion (1.411% vs 15.453% and 10.108%, respectively). All Protocol K samples KPT1, KPT2, and KNPT1 exhibited quite similar proportions of reads with aDNA characteristic damage (5.742%, 7,500%, and 4.321%, respectively) (Table S1).

Negative controls for both DNA isolation protocols and amplification blanks were sequenced to an average of 150,000 reads each. Percentage of human genome aligning reads ranged from 0.780% to 8.652% of endogenous reads for Protocol V blanks and 0.071% for Protocol K blank (Table 2). Almost all processed negative extraction controls of Protocol V (7.143% and 9.525%) had several times higher human-aligning duplication ratio than associated bone and tooth samples. Proportion of duplicates was especially high for Protocol K blank, reaching 57.191% (Table S1). None of the negative controls had ancient damage patterns (Fig. S2). Proportion of damaged human and mitochondrial genome reference aligning reads varied between 0% and 0.757% for Protocol V blanks. For Protocol K blank, the proportion of damaged reads was 1.020% (Table S1).

**TABLE 2:** Assigned read proportions for negative controls by MALT and Kraken2 metagenomic analysis tools^*a*^

Code	Number of merged and trimmed reads	Total Kraken2 assigned reads	Proportion of Kraken2 assigned reads	Total MALT assigned reads	Proportion of MALT assigned reads
VNCE1	15950	4,329	27.141%	3,210	20.125%
VNCL1	641	57	8.892%	21	3.276%
VNCE2	21372	5,377	25.159%	3,742	17.509%
VNCL2	235	26	11.064%	6	2.553%
KNC1	831860	724,897	87.142%	3,547	0.426%

^*a*^ Full Kraken2 and MALT results are available in Supplementary materials (Tables S2 to S5).

### Comparative analysis of metagenomic profiles

Negative control samples, in general, had a low number of total microorganisms-assigned reads, and the analysis indicated low complexity for library construction negative controls. Information about the microbial reference genomes to which sequencing reads were aligned is summarized in Tables S2 to S5. Microbial-aligned read count for library construction negative controls was even lower (Table 2). Metagenomic profiles of Protocol K and Protocol V negative controls differed greatly (Fig. S6).

### Microbiome profiling results based on Kraken2 analysis

**Fig 3:**
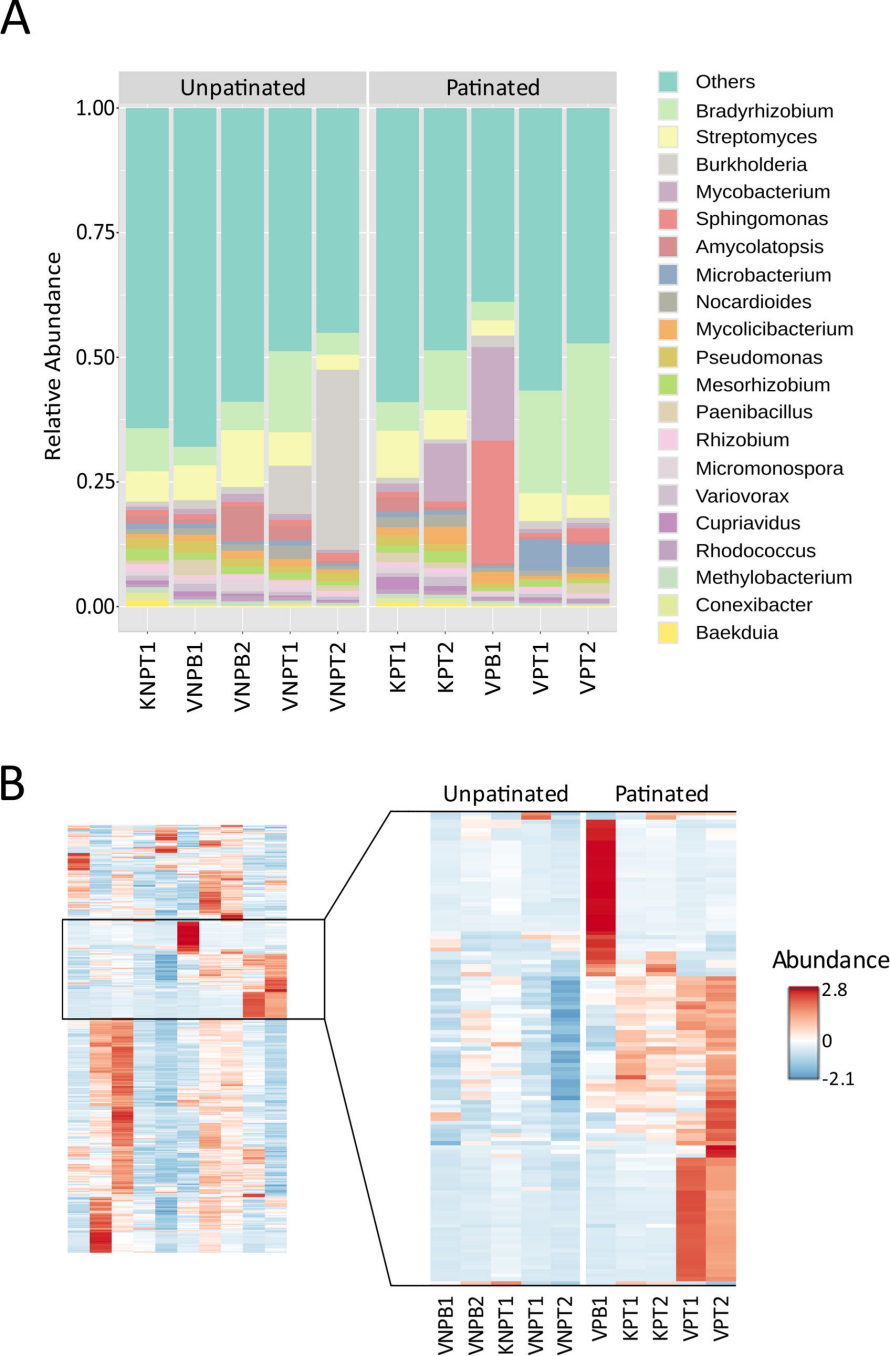
(**A**) Relative abundance stack plot of all analyzed samples of this study. Only the 20 most abundant genera are displayed. (**B**) Heatmap of bacterial genera abundance of all samples. A zoomed-in section shows 112 genera which were more abundant in analyzed samples. A full list of microbial genera identified in samples in identical order can be found in Table S11.

To highlight the most represented genera according to *Kraken2*, a shortlist was made with top 10 genera of each sample (Tables S6 and S7). Relative abundance analysis indicated high overall representation of *Bradyrhizobium* and *Streptomyces* aligning reads in all analyzed samples (Fig. 3A). Also, both patinated and unpatinated samples with a lesser prevalence did include genera like *Paenibacillus*, *Mycobacterium, Agromyces,* and *Nocardioides*.

More specifically, when comparing *Kraken2* shortlists of patinated Protocol V tooth samples (VPT1 and VPT2) with unpatinated samples (VNPT1 and VNPT2), both VPT1 and VPT2 had high *Microbacterium* and *Salinibacterium* genera representation, while microbial genera found to be most abundant solely in unpatinated samples (VNPT1, VNPT2, VNPB1, and VNPB2) were *Amycolatopsis*, *Pseudomonas*, *Micromonospora,* and *Variovorax*.

On the other hand, no clear trend dependent on presence of patination could be observed for Protocol K tooth samples (KPT1, KPT2, KNPT1): universally present in all three sample shortlists were *Bradyrhizobium*, *Streptomyces*, *Sphingomonas*, and *Pseudomonas* genera (Table S7). Patination-specific genera found in Protocol K samples were *Nocardioides*, *Mycobacterium*, and *Paenibacillus*.

Overall, significant variations of microbiome profile were observed for patinated samples. Increase of *Cupriavidus* aligned reads was observed for severely patinated samples KPT2 and VPB1. Additionally, VPT1 and VPT2 showed increased proportions of *Microbacterium*, and *Amycolatopsis* proportion was greater in KPT1 in comparison to other patinated samples.

For a single patinated bone sample, VPB1 metagenomic analysis showed significantly increased amounts of *Sphingomonas* reads (Fig. 3A). The assigned read proportions reached 28.727% on genus and 36.891% on family levels (Table S8), which is well reflected also by *Kraken2* top 10 shortlist (Table S7) of most abundant genera, which includes four genera of *Sphingomonadaceae*: *Sphingomonas*, *Sphingobium*, *Sphingopyxis*, and *Rhizorhabdus*.

Heatmap analysis revealed more than 100 bacterial genera with increased abundance of reads aligned for patinated samples in comparison to unpatinated ones (Fig. 3B).

**Fig 4:**
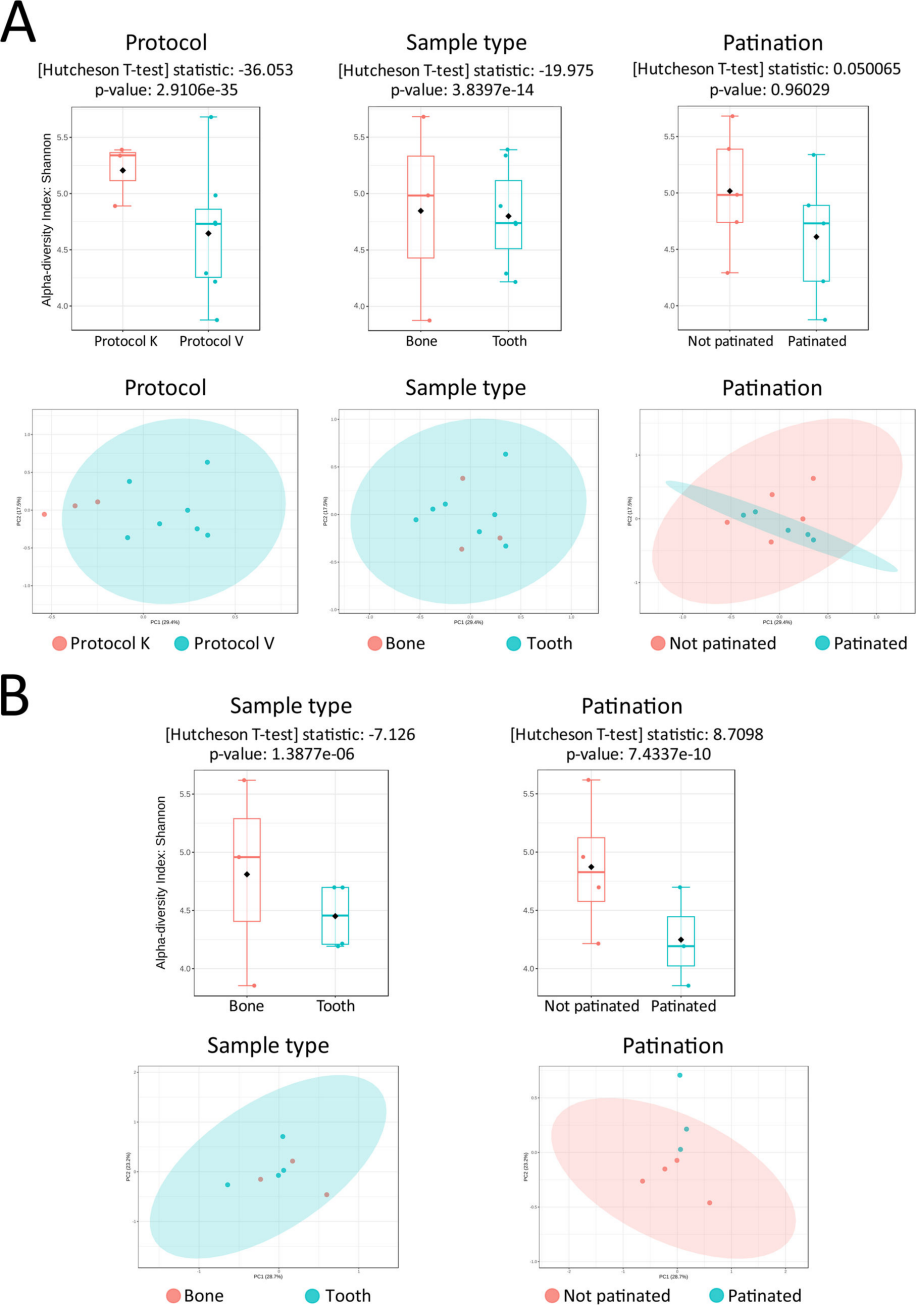
(**A**) Top: alpha diversity (Shannon index and Hutcheson’s *t*-test/ANOVA) analysis of all three variables used in this study. The *P*-value of protocol choice as a variable is extremely low (*P*-value: 2.9106e-35) and indicates strong statistical significance for protocol affecting comparison of diversity. Bottom: PCA of all three variables and their effects on the clustering of samples. (**B**) Top: alpha diversity (Shannon index and Hutcheson’s *t*-test/ANOVA) analysis of sample type and patination as variables. Both variables (Sample type *P*-value: 1.3877e-06; Patination *P*-value: 7.4337e-10) show statistically significant effect on diversity of metagenomic profiles of analyzed samples. Bottom: PCA of only Protocol V samples and sample type or patination effects on the clustering of samples.

Alpha diversity analysis performed between such variables like the protocol used, sample type, and patination using Shannon index and Hutcheson’s *t*-test/analysis of variance (ANOVA) (Fig. 4A) revealed that the protocol had the highest impact on the difference in alpha diversity of the metagenomic profiles. Additionally, the sample type had affected metagenomic profiles statistically significantly. Patination had no statistically significant effect on the difference between metagenomic composition.

Principal component analysis (PCA) analysis of metagenomic profiles was performed separately for all variables (Fig. 4A). Distinct clustering could be observed only in DNA isolation protocol-based and patination-based PCA plots.

Furthermore, taking into account the statistically significant difference between the protocols used, effect of patination, and sample type, additional analysis was performed for protocol V samples separately (Fig. 4B). For alpha-diversity, *P*-values for both sample type and the presence/absence of patination were lower than 0.05, and patination as a variable had a higher impact than sample type on statistical significance of comparison of diversity. PCA analysis of only Protocol V samples showed even dispersal regardless of sample type, while for patination as variable, areas of dispersal of patinated and unpatinated samples did not overlap (Fig. 4B).

### Microbiome profiling results based on MALT analysis

In order to assess the robustness of *Kraken2* findings, an additional metagenomic analysis tool was used—MALT, which aligns NGS reads to a DNA database using the BLASTN algorithm.

Based on the fact that MALT assigns reads to specific species level reference (Table S9), top 20 bacterial references were picked for each of the analyzed samples and compared among Protocol V and Protocol K sample groups (Table S10). Among all Protocol V samples (VPT1, VPT2, VNPT1, VNPT2, VPB1, VNPB1, and VNPB2), most of the aligned reads were aligned to *Bradyrhizobium* and *Burkholderia* genera, which differs from *Kraken2* results (*Bradyrhizobium* and *Streptomyces*).

When comparing patinated and unpatinated tooth samples (VPT1, VPT2, VNPT1, and VNPT2), only two other genera could be identified in both patinated and unpatinated sample data*—Agromyces* and *Cutibacterium*.

Top 20 reference shortlists for patinated tooth samples VPT1 and VPT2 differed from unpatinated shortlists by having more similar bacteria. Only one reference between VPT1 and VPT2 each did not overlap between the samples. Both samples shared *Microbacterium*, *Chryseolinea*, *Glaciihabitans*, *Salinibacterium*, *Marisediminicola*, *Clavibacter,* and *Protaetiibacter* genera as the most represented.

All samples of bone origin (VPB1, VNPB1, and VNPB2) exhibited higher microbial reference diversity and were less similar among each other than the tooth samples.

In accordance to the Kraken2 results, a very high prevalence of *Sphingomonas* genera was observed for VPB1, which is further supported by total percentage of *Sphingomonas* genus aligning reads reaching 22.37% and on *Sphingomonadaceae* family level 25.98% of all *MALT* assigned reads. *Sphingomonas* could be also detected in VNPT2 shortlist but to a much lesser proportion (1.54%). Additionally, for the VPB1 sample, a quarter of top 20 references were of *Mycobacterium* genus (Table S10).

Only unpatinated Protocol V samples did possess enough *Amycolatopsis* aligning reads to be included in the top 20. In the patinated samples, this particular genus did not even reach the top 100 references.

Similarly to *Kraken2* analysis, Protocol K patinated (KPT1 and KPT2) and unpatinated (KNPT1) tooth samples did not exhibit pronounced differences. The sample reference shortlists for all three samples included *Bradyrhizobium* and *Conexibacter* genera reads, and other genera overlapped between the samples without a clear trend like the one observed for Protocol V samples. KPT1, a partially patinated sample, had four *Amycolatopsis* references in its shortlist. KPT2, which was a severely patinated tooth sample, possessed a wide array of references of *Mycobacterium* genus similarly to VPB1 and one *Chryseolinea soli* reference, which was identified for all Protocol V tooth samples.

## DISCUSSION

### Human DNA preservation

In this study, aDNA authentication analysis revealed that most samples without patination possessed convincing cytosine deamination complementary frequency patterns at the 3′ end of the sequences, indicating the authenticity of human genome aligning reads (29). All but one patinated samples, however, did not exhibit these damage patterns convincingly in our study, suggesting lower endogenous DNA preservation; the only sample displaying pronounced authentic aDNA characteristic damage was sample KPT1, which was obtained from a partially patinated tooth (Fig. 1). As it was the least patinated out of all analyzed samples, it is possible that mineralization which occurs in the presence of copper-containing alloys and chemical contamination of the tissue (1) had occurred only partially, resulting in either better preservation of endogenous human aDNA, or less pronounced interference with aDNA analysis techniques.

Interestingly, there were also differences in the number of the obtained human aligning reads from the patinated teeth, which was 2 to 200× lower than for the unpatinated ones, and in three samples even not meeting the suggested threshold for proper aDNA authentication (44). This observation is in accordance with modern DNA amplification results in forensic contexts, which is shown to be problematic due to the damage caused to the DNA molecule by binding of metal ions, which can lead to single and double-stranded breaks of the molecule (8, 45). Such intensive molecular damage during early decomposition stages could enhance the rate of normal DNA degradation process, possibly leading to poorer human DNA preservation, although we did not observe shorter DNA fragments in patinated samples (Table S1).

Additionally, the proportion of quality filtered reads for all patinated samples was smaller than the proportion of quality filtered reads for negative controls processed in the same batch, which might indicate presence of PCR inhibitory factors in the aDNA extract.

Duplication ratio of human-aligning reads in analyzed samples is low, but highly variable. For Protocol V, tooth samples duplication decreased more than 10-fold when comparing patinated versus unpatinated samples. A similar trend could be observed for Protocol K samples, with a decrease in duplication ratio, relating to increased patination intensity. This could be explained by a very low initial human DNA proportion in DNA extract, combined with insufficient number of PCR cycles (*N* = 10) to increase human DNA proportion in the sample to as substantial amounts as for unpatinated samples. Based on the damage profile of VPB1 (Fig. 2; Fig. S4), indicating possible modern contamination, comparison of duplication rates of patinated and unpatinated bone samples could be problematic.

Our reported proportions of damaged human genome and mtDNA reference aligning reads, based on PMD score ≥ 3, are low (30); however, at least partially, it could be explained by methodological challenges leading to the theoretical aDNA losses. In particular, the original polymerase in the kit used was unable to process the uracils, thus, theoretically, decreasing the proportion of aDNA Nevertheless, we can see a strong contrast in aDNA proportion values for patinated and unpatinated Protocol V bone samples. As the damage patterns have been previously identified for another patinated sample (KPT1), it is possible that its inconclusive G to A transition frequency pattern at the 3′ end has been caused by the persisting small proportion (1.411%) of damaged authentic aDNA; therefore, such comparison is permissible. When excluding VNPT2, which displayed an unconvincing damage pattern, indicating possible modern contamination, for Protocol V tooth samples, an almost twofold difference in damaged read proportion between VNPT1 (4.993%) and VPT2 (2.721%) can be observed, following the same trend. No similar relation could be observed for Protocol K samples, indicating the importance of methodological nuances.

### Microbiome analysis

Both *Kraken2* and *MALT* analysis indicated a strong presence of the *Bradyrhizobium* genus in all analyzed samples, which is known for its nitrogen-fixing abilities and is a common genus among soil bacteria (46). According to *Kraken2* results, the other universally detected genus was *Streptomyces*, which is a very broad genus with more than 800 described species and present in a wide range of terrestrial and marine environments (47–49).

According to *MALT* analysis, the other genus found in all analyzed samples was *Burkholderia*, which is a very heterogeneous genus, containing human and animal pathogens, rhizobacteria, and soil bacteria (50). When analyzed to lower taxa levels, most of *Burkholderia* reads were aligned to *Burkholderia contaminans*, which is a part of *Burkholderia cepacia complex*—a group of opportunistic pathogens, isolated from immunocompromised patients with cystic fibrosis (51). This does not indicate possible infection and more likely could be attributed to poorer representation of soil bacteria genera in reference databases, as those are, unfortunately, not as described as pathogens of the same genus.

Regardless of patination, occasional presence of *Agromyces*, *Nocardioides*, *Mycobacterium*, *Paenibacillus,* and *Cutibacterium* was identified. *Agromyces*, just as *Nocardioides* and *Paenibacillus*, are predominantly associated with soil environments (52–54). Although the *Mycobacterium* genus does include well-known pathogens like *Mycobacterium tuberculosis*, great diversity of *Mycobacterium* genus bacteria can be commonly found in terrestrial environments, many of which are still undescribed (55). While possible infection with *M. tuberculosis* or other pathogenic nontuberculous mycobacteria cannot be completely ruled out without greater sequencing efforts or targeted capture which would allow aDNA damage assessment, current *MALT* read count distribution among references of *Mycobacterium* genus indicates alignment to non-tuberculous mycobacteria, which have been previously isolated from a variety of environmental reservoirs, including soil (56).

*Cutibacterium acnes* from the *Cutibacterium* genus is one of the most common of human skin bacterial species (57). This strongly indicates persistence of modern contaminants in analyzed samples even after the decontamination step. Due to the usage of negative controls which account for possible lab contamination, it is plausible that these contaminants could have gotten deeper in the sampled tissue than just on the surface during the handling and storage period between excavation and clean lab preparations.

In patinated samples, the microbiome analysis revealed the increased abundance of microbial species that could tolerate metals and salt. It is known that several laboratory microbial contaminants are also metal- and bactericide-resistant (58). However, even after the read filtering step which excluded laboratory contamination from the data sets of our samples based on the genetic material from the negative controls, we still saw these reads persisting. Specifically, among the Protocol V samples, seven patination-specific genera were identified: *Chryseolinea*, *Glaciihabitans*, *Marisediminicola*, *Clavibacter*, *Protaetiibacter*, *Microbacterium,* and *Salinibacterium*.

From those, only the last two were supported by both metagenomic analysis methods. *Microbacterium*, which is often considered to be a laboratory contaminant (58), is also an environmental bacteria, present in soils and resistant to heavy metals (59). *Salinibacterium*, on the other hand, is known to be salt-tolerant (60).

Other genera identified are usually found in soil (61–64), larva gut (65, 66), plants (as pathogens) (67), aquatic sediments, glacial ecosystems, and marine intertidal zones (68–70).

Bacteria of the *Sphingomonas* genus are a common gram-negative, chemoheterotrophic, strictly aerobic soil bacteria with biodegradative capabilities including organometallic compounds (71–74). Therefore, the increased read percentage of soil bacteria able to withstand the properties of copper-containing alloys is not surprising. Although the presence of this bacterial genus could be seen in other samples as well (Tables S8 and S9), abundance of the *Sphingomonadaceae* family reads in VPB1 probably indicates better compatibility of particular genera with severely patinated environments than any other found genera.

An interesting observation was the decrease of *Amycolatopsis* genus reads in the patinated samples. *Amycolatopsis* is a soil bacteria genus (75), whose secondary metabolites have antibacterial properties, and it is used in antibiotic production and heavy metal immobilization (76, 77). A possible explanation for this observation could be that *Amycolatopsis* aligning reads did not originate from *Amycolatopsis tucumanensis*, *Amycolatopsis* sp. GT6, *Amycolatopsis* sp. GT15, or *Amycolatopsis* sp. GT39, which are the only species in *Amycolatopsis* genus resistant to heavy metals (76, 78). This is also supported by our results of lower taxa level analysis with *MALT. Variovorax* genus bacteria is widely found in soil and aquatic environments (79). *Micromonospora*, in addition to previously mentioned, has also been found in association with plants (80), while *Pseudomonas* has also been identified in clinical environments (81).

Overall results of *Kraken2* and *MALT* analysis of Protocol K samples did not exhibit such clear trends, most probably due to the small sample size as it was impossible to distinguish whether presence of certain genus in the metagenomic profile was patination or sample type affected or just characteristic of the sampled tooth specifically. Similarly to the Protocol V samples, *Bradyrhizobium* presence was observed in all processed Protocol K samples regardless of metagenomic alignment tool. *Kraken2* analysis reveals additional universally present already discussed genera *Pseudomonas*, *Sphingomonas*, and *Streptomyces*, while *MALT* analysis results indicated the additional presence of *Conexibacter*, which is associated with isolated terrestrial environments (82).

Of three Protocol K samples, only KPT2 had a patination-specific *Chryseolinea* genus aligning reads, which had been found in Protocol V patinated tooth samples; all patination-specific genera identified by *Kraken2* analysis were patination non-specific in the case of Protocol V*—Nocardioides*, *Mycobacterium*, and *Paenibacillus,* while *MALT* analysis did not reveal any differences.

Interestingly, partially patinated tooth sample KPT1 had a considerable proportion of reads attributed to *Amycolatopsis* genus, as supported by both *Kraken2* and *MALT* analysis, and showed overall higher similarity to the unpatinated sample.

### Variability assessment of metagenomic diversity

Relative microbial abundance analysis followed previously discussed trends. Most of the samples that exhibit a similar trend (VNPT1 and VNPT2 and VPT1 and VPT2) have all three variables (protocol, sample type, and presence of patination) in common. The only exception is KPT2 and VPB1, for which the only common variable is patination where both samples have an increased *Cupriavidus* proportion—genus which is not only resistant to but also its growth is stimulated by copper (83, 84).

As indicated by the heatmap analysis, there is a trend for reads of patinated sample origin to map specifically to a certain heterogeneous group of bacterial genera. We could not find another unifying resistance factor for these genera, as there is a lack of uniform research about their growth, tolerance, resistance, and other significant aspects which could lead to such results. From among 112 genera observed, we found bacteria within these genera with described halophilic and alkalophilic properties and resistance to bactericides as well as genera without these properties or those not being described (85).

The majority of microbial genera assessed were associated with soil and aquatic environments, and to a lesser degree, ecosystems like tidal flats and swamps. These results, in addition to observations made from *MALT* and *Kraken2* metagenomic profiles, are consistent with the location of the burial site being located at the floodplain of Daugava, where terrestrial and aquatic environments overlap.

An interesting observation in this context is the presence of marine bacteria aligning reads for patinated samples, because of the substantial distance (~ 90 km [~ 60 mi]) from the archaeological site to the coastal line. Due to the stark change in abundance when compared with unpatinated samples, a plausible explanation for this would be an increased presence of copper or other salts in the alloy of the patinated decoration. Analysis of chemical composition of alloys and supernatants from the preincubation step could give more precise insight into the chemical causative agent for this phenomenon.

The *p*-value of alpha diversity analysis very strongly indicated the immense effect of extraction protocol on difference between metagenomic profiles. The 15-minute pre-incubation step, where supernatant was not used for DNA extraction, could have removed some bacterial, burial soil-related background which persisted in Protocol K samples. Separate PCA analysis with “Protocol” as the variable also showed Protocol K samples clustering away from the Protocol V distinctly.

Thus, we also analyzed Protocol V samples separately, aiming to exclude this protocol-related variability. Limited PCA analysis of Protocol V samples revealed that the patinated sample dispersal area did not overlap with un-patinated samples. Also, repeated and limited alpha diversity analysis supported not only sample type as an influential variable affecting the composition of metagenomic profile (86) but also the presence of patination, with a statistical significance.

### Study limitations

This study has several limitations. The greatest limitation for this study was the poor preservation of the analyzed osteological material in general. Further limitations were the low number of analyzed samples and various degrees of copper patination, as well as differences in DNA extraction protocols used. In addition, all samples within this study have been subjected to a significant loss of information due to the commercial kit polymerase used for library construction. The inability of the polymerase to process the uracils has led to not only distorted damage profiles, exhibiting only cytosine deamination complementary patterns at the 3′ end of the sequences but also decreasing the theoretical proportion of authentic human aDNA. Therefore, caution is advised in comparing these results directly with data generated outside this particular study. Unfortunately, these limitations could not be addressed during the study because of the limited number of archaeological samples available, as well as financial limitations. However, despite some shortcomings, we were able to obtain results that provided some novel insights for future aDNA studies in patinated osteological material.

### Conclusions

Our results suggest that patinated teeth and bones may not be an optimal source of human aDNA. The endogenous DNA preservation for patinated bones varied according to the degree of patination which is closely associated with mineralization. Although patinated bones and teeth seem to have better visual preservation due to mineralization, our data suggest that bone tissue with copper patina could have a very low endogenous DNA proportion, while tooth samples with less patination might have not mineralized to an extent where the endogenous DNA had been degraded completely.

Metagenomic profile comparison between the patinated and unpatinated samples revealed several differences in the abundance of soil and aquatic bacteria, with some resistant to heavy metals or salt present more often, and the impact of patination on microbial profiles being statistically significant.

With our current knowledge of the low endogenous DNA percentage in archaeological samples, deeper sequencing should be considered, as the small number of human and bacteria aligning reads could not be confirmed as authentic or as modern contamination; thus definite conclusions were not possible.

Future research objectives should consist of using a larger sample set from several archaeological sites and energy-dispersive X-ray spectroscopy to assess the chemical composition of the mineralized hard tissue samples.

## Data Availability

Raw sequencing reads have been submitted to the European Nucleotide Archive, project accession PRJEB79566.

## SUPPLEMENTAL MATERIAL

Supplemental figures spectrum.02705-24-s0001.pdf Figures S1 to S6.

Supplemental tables spectrum.02705-24-s0002.xlsx Tables S1 to S11.

ASM does not own the copyrights to Supplemental Material that may be linked to, or accessed through, an article. The authors have granted ASM a non-exclusive, world-wide license to publish the Supplemental Material files. Please contact the corresponding author directly for reuse.

## References

[1] Oddy A, Scott DA. 2002. Copper and bronze in art: corrosion, colorants, conservation. Stud Conserv 47:277. doi:10.2307/1506788

[2] Tylecote RF. 2002. A history of metallurgy. Many Publishing for Institute of Materials.

[3] Scott DA, Taniguchi Y, Koseto E. 2001. The verisimilitude of verdigris: a review of the copper carboxylates. Studies in Conservation 46:73–91. doi:10.1179/sic.2001.46.Supplement-1.73

[4] Gillard RD, Hardman SM, Thomas RG, Watkinson DE. 1994. The mineralization of fibres in burial environments. Studies in Conservation 39:132–140. doi:10.1179/sic.1994.39.2.132

[5] Jia R, Zheng H, Chen H, Feng M, Jiao J, Kang X, Yu J, Wang B, Zhang Z, Zhou Y, Peng Z. 2024. Archaeological textiles preserved by copper mineralization. Herit Sci 12:312. doi:10.1186/s40494-024-01418-8

[6] Robbiola L, Moret P, Lejars T. 2011. A case study of arthropods preserved on archaeological bronzes—micro‐archaeological investigation helps reconstructing past environments. Archaeometry 53:1249–1256. doi:10.1111/j.1475-4754.2011.00607.x

[7] Bonsu M, Higgins D, Austin J. 2023. Zinc, not copper, is the major contributor to DNA degradation and PCR inhibition in DNA samples contaminated with brass. Available from: 10.2139/ssrn.4412717

[8] Czado N, Houston R, Hughes S. 2022. Comparison of metal ions recovered during DNA analysis of brass ammunition and effects of copper and zinc ions on DNA profiling. Forensic Sci Int Genet Suppl Ser 8:162–164. doi:10.1016/j.fsigss.2022.10.021

[9] Grass G, Rensing C, Solioz M. 2011. Metallic copper as an antimicrobial surface. Appl Environ Microbiol 77:1541–1547. doi:10.1128/AEM.02766-10 21193661 PMC3067274

[10] Timoncini A, Costantini F, Bernardi E, Martini C, Mugnai F, Mancuso FP, Sassoni E, Ospitali F, Chiavari C. 2022. Insight on bacteria communities in outdoor bronze and marble artefacts in a changing environment. Sci Total Environ 850:157804. doi:10.1016/j.scitotenv.2022.157804 35932861

[11] Urtāns V. 1965. Izrakumi Lejasbitēnu un Lejasziedu apmetnēs, Kalnaziedu pilskalnā un Lejasbitēnu kapulaukā [Archaeological excavations in Lejasbitēni and Lejasziedi settlements, Kalnaziedi hillfort, and Lejasbitēni cemetery]. Zinātniskās atskaites sesijas referātu tēzes par arheologu, etnogrāfu un folkloristu 1964.gada ekspedīcijām, Rīga, p 21–24

[12] Pētersone-Gordina E, Gerhards G, Vilcāne A, Millard AR, Moore J, Ķimsis J, Ranka R. 2022. Diet and social status in the Lejasbitēni Iron Age population from Latvia. J Archaeol Sci 44:103519. doi:10.1016/j.jasrep.2022.103519

[13] Buikstra JE, Ubelaker DH. 1994. Standards for data collection from human skeletal remains, p 44. In Archeological survey research seminar series

[14] AlQahtani SJ, Hector MP, Liversidge HM. 2010. Brief communication: the London atlas of human tooth development and eruption. Am J Phys Anthropol 142:481–490. doi:10.1002/ajpa.21258 20310064

[15] Schour I, Massler M. 1941. The development of the human dentition. J Am Dent Assoc (Ed Ital) 28:1153.

[16] Fulton TL, Shapiro B. 2019. Setting up an ancient DNA laboratory, p 1–13. In Shapiro B, Barlow A, Heintzman DP, Hofreiter M, Paijmans A, Soares R (ed), Ancient DNA, methods in molecular biology. Springer, New York, New York, NY. 10.1007/978-1-4939-9176-1_1 30875038

[17] The Supreme Council of the Republic of Latvia. 1992. “On the protection of the body of deceased human beings and the use of human tissues and organs in medicine”. Available from: https://likumi.lv/ta/en/en/id/62843-on-the-protection-of-the-body-of-deceased-human-beings-and-the-use-of-human-tissues-and-organs-in-medicine

[18] Keyser-Tracqui C, Ludes B. 2005. Methods for the study of ancient DNA, p 253–264. In Forensic DNA typing protocols. Humana Press, New Jersey. 10.1385/1-59259-867-6:253 15570113

[19] Velsko I, Skourtanioti E, Brandt G. 2020. Ancient DNA extraction from skeletal material v1. Protocols.io. doi:10.17504/protocols.io.baksicwe

[20] Dabney J, Knapp M, Glocke I, Gansauge M-T, Weihmann A, Nickel B, Valdiosera C, García N, Pääbo S, Arsuaga J-L, Meyer M. 2013. Complete mitochondrial genome sequence of a Middle Pleistocene cave bear reconstructed from ultrashort DNA fragments. Proc Natl Acad Sci U S A 110:15758–15763. doi:10.1073/pnas.1314445110 24019490 PMC3785785

[21] Blevins K, Plug JH, Pearson J, Molist M, Bach A, Fernández Domínguez E. 2023. A test of pretreatment and DNA extraction methods for uncovering endogenous content in Neolithic human remains from the Near East. 10th Meeting of the International Society for Biomolecular Archaeology (ISBA) New Horizons in Biomolecular Archaeology Abstract Book, p 89

[22] Gamba C, Hanghøj K, Gaunitz C, Alfarhan AH, Alquraishi SA, Al-Rasheid KAS, Bradley DG, Orlando L. 2016. Comparing the performance of three ancient DNA extraction methods for high-throughput sequencing. Mol Ecol Resour 16:459–469. doi:10.1111/1755-0998.12470 26401836

[23] Yang DY, Eng B, Waye JS, Dudar JC, Saunders SR. 1998. Technical note: improved DNA extraction from ancient bones using silica-based spin columns. Am J Phys Anthropol 105:539–543. doi:10.1002/(SICI)1096-8644(199804)105:4<539::AID-AJPA10>3.0.CO;2-1 9584894

[24] Bolger AM, Lohse M, Usadel B. 2014. Trimmomatic: a flexible trimmer for Illumina sequence data. Bioinformatics 30:2114–2120. doi:10.1093/bioinformatics/btu170 24695404 PMC4103590

[25] Peltzer A, Jäger G, Herbig A, Seitz A, Kniep C, Krause J, Nieselt K. 2016. EAGER: efficient ancient genome reconstruction. Genome Biol 17:60. doi:10.1186/s13059-016-0918-z 27036623 PMC4815194

[26] Oliva A, Tobler R, Llamas B, Souilmi Y. 2021. Additional evaluations show that specific BWA-aln settings still outperform BWA-mem for ancient DNA data alignment. Ecol Evol 11:18743–18748. doi:10.1002/ece3.8297 35003706 PMC8717315

[27] Picard Toolkit. 2019. Broad institute, GitHub repository. Available from: https://broadinstitute.github.io/picard

[28] Danecek P, Bonfield JK, Liddle J, Marshall J, Ohan V, Pollard MO, Whitwham A, Keane T, McCarthy SA, Davies RM, Li H. 2021. Twelve years of SAMtools and BCFtools. Gigascience 10:giab008. doi:10.1093/gigascience/giab008 33590861 PMC7931819

[29] Jónsson H, Ginolhac A, Schubert M, Johnson PLF, Orlando L. 2013. mapDamage2.0: fast approximate Bayesian estimates of ancient DNA damage parameters. Bioinformatics 29:1682–1684. doi:10.1093/bioinformatics/btt193 23613487 PMC3694634

[30] Skoglund P, Northoff BH, Shunkov MV, Derevianko AP, Pääbo S, Krause J, Jakobsson M. 2014. Separating endogenous ancient DNA from modern day contamination in a Siberian Neandertal. Proc Natl Acad Sci U S A 111:2229–2234. doi:10.1073/pnas.1318934111 24469802 PMC3926038

[31] Moilanen U, Kirkinen T, Saari N-J, Rohrlach AB, Krause J, Onkamo P, Salmela E. 2022. A woman with a Sword? – Weapon grave at Suontaka Vesitorninmäki, Finland. Eur J archaeol 25:42–60. doi:10.1017/eaa.2021.30

[32] Renaud G, Slon V, Duggan AT, Kelso J. 2015. Schmutzi: estimation of contamination and endogenous mitochondrial consensus calling for ancient DNA. Genome Biol 16:224. doi:10.1186/s13059-015-0776-0 26458810 PMC4601135

[33] Fu Q, Mittnik A, Johnson PLF, Bos K, Lari M, Bollongino R, Sun C, Giemsch L, Schmitz R, Burger J, Ronchitelli AM, Martini F, Cremonesi RG, Svoboda J, Bauer P, Caramelli D, Castellano S, Reich D, Pääbo S, Krause J. 2013. A revised timescale for human evolution based on ancient mitochondrial genomes. Curr Biol 23:553–559. doi:10.1016/j.cub.2013.02.044 23523248 PMC5036973

[34] Korneliussen TS, Albrechtsen A, Nielsen R. 2014. ANGSD: analysis of next generation sequencing data. BMC Bioinformatics 15:356. doi:10.1186/s12859-014-0356-4 25420514 PMC4248462

[35] Moreno-Mayar JV, Korneliussen TS, Dalal J, Renaud G, Albrechtsen A, Nielsen R, Malaspinas A-S. 2020. A likelihood method for estimating present-day human contamination in ancient male samples using low-depth X-chromosome data. Bioinformatics 36:828–841. doi:10.1093/bioinformatics/btz660 31504166 PMC8215924

[36] Shen W, Le S, Li Y, Hu F. 2016. SeqKit: a cross-platform and ultrafast toolkit for FASTA/Q file manipulation. PLOS ONE 11:e0163962. doi:10.1371/journal.pone.0163962 27706213 PMC5051824

[37] Gordon A. 2010. FASTX-toolkit. Available from: http://hannonlab.cshl.edu/fastx_toolkit/. Retrieved 20 Apr 2024.

[38] Huson DH. 2022. Megan alignement tool. Available from: https://software-ab.cs.uni-tuebingen.de/download/malt/welcome.html. Retrieved 20 Apr 2024.

[39] Wood DE, Salzberg SL. 2014. Kraken: ultrafast metagenomic sequence classification using exact alignments. Genome Biol 15:R46. doi:10.1186/gb-2014-15-3-r46 24580807 PMC4053813

[40] McKnight DT, Huerlimann R, Bower DS, Schwarzkopf L, Alford RA, Zenger KR. 2019. microDecon: a highly accurate read‐subtraction tool for the post‐sequencing removal of contamination in metabarcoding studies. Environmental DNA 1:14–25. doi:10.1002/edn3.11

[41] Lu J, Breitwieser FP, Thielen P, Salzberg SL. 2017. Bracken: estimating species abundance in metagenomics data. PeerJ Comput Sci 3:e104. doi:10.7717/peerj-cs.104 PMC12016282 40271438

[42] Chong J, Liu P, Zhou G, Xia J. 2020. Using MicrobiomeAnalyst for comprehensive statistical, functional, and meta-analysis of microbiome data. Nat Protoc 15:799–821. doi:10.1038/s41596-019-0264-1 31942082

[43] Dhariwal A, Chong J, Habib S, King IL, Agellon LB, Xia J. 2017. MicrobiomeAnalyst: a web-based tool for comprehensive statistical, visual and meta-analysis of microbiome data. Nucleic Acids Res 45:W180–W188. doi:10.1093/nar/gkx295 28449106 PMC5570177

[44] Warinner C, Herbig A, Mann A, Fellows Yates JA, Weiß CL, Burbano HA, Orlando L, Krause J. 2017. A robust framework for microbial archaeology. Annu Rev Genomics Hum Genet 18:321–356. doi:10.1146/annurev-genom-091416-035526 28460196 PMC5581243

[45] Anastassopoulou J. 2003. Metal–DNA interactions. J Mol Struct 651–653:19–26. doi:10.1016/S0022-2860(02)00625-7

[46] Jordan DC. 1982. NOTES: transfer of Rhizobium japonicum Buchanan 1980 to Bradyrhizobium gen. nov., a genus of slow-growing, root nodule bacteria from leguminous plants. Int J Syst Bacteriol 32:136–139. doi:10.1099/00207713-32-1-136

[47] Alam K, Mazumder A, Sikdar S, Zhao Y-M, Hao J, Song C, Wang Y, Sarkar R, Islam S, Zhang Y, Li A. 2022. Streptomyces: the biofactory of secondary metabolites. Front Microbiol 13:968053. doi:10.3389/fmicb.2022.968053 36246257 PMC9558229

[48] Aryal S, Neupane L, Adhikari R, Regmi B, Koirala N, Joshi DR. 2021. Novel Streptomyces sp. reported in 2018: a meta-analysis. Anti Infect Agents 19:3–14. doi:10.2174/2211352518666200423083354

[49] Kemung HM, Tan LT-H, Khan TM, Chan K-G, Pusparajah P, Goh B-H, Lee L-H. 2018. Streptomyces as a prominent resource of future Anti-MRSA drugs. Front Microbiol 9:2221. doi:10.3389/fmicb.2018.02221 30319563 PMC6165876

[50] Bothe H, Ferguson SJ, Newton WE. 2007. Biology of the nitrogen cycle. Elsevier, Amsterdam Boston.

[51] Mahenthiralingam E, Urban TA, Goldberg JB. 2005. The multifarious, multireplicon Burkholderia cepacia complex. Nat Rev Microbiol 3:144–156. doi:10.1038/nrmicro1085 15643431

[52] Ash C, Priest FG, Collins MD. 1993. Molecular identification of rRNA group 3 bacilli (Ash, Farrow, Wallbanks and Collins) using a PCR probe test. Proposal for the creation of a new genus Paenibacillus. Antonie Van Leeuwenhoek 64:253–260. doi:10.1007/BF00873085 8085788

[53] Gledhill WE, Casida LE Jr. 1969. Predominant catalase-negative soil bacteria. III. Agromyces, gen. n., microorganisms intermediary to Actinomyces and Nocardia. Appl Microbiol 18:340–349. doi:10.1128/am.18.3.340-349.1969 16349860 PMC377982

[54] Prauser H. 1976. Nocardioides, a new genus of the order Actinomycetales. Int J Syst Bacteriol 26:58–65. doi:10.1099/00207713-26-1-58

[55] Walsh CM, Gebert MJ, Delgado-Baquerizo M, Maestre FT, Fierer N. 2019. A global survey of mycobacterial diversity in soil. Appl Environ Microbiol 85:e01180-19. doi:10.1128/AEM.01180-19 31253672 PMC6696970

[56] Mercaldo RA, Marshall JE, Cangelosi GA, Donohue M, Falkinham JO 3rd, Fierer N, French JP, Gebert MJ, Honda JR, Lipner EM, Marras TK, Morimoto K, Salfinger M, Stout J, Thomson R, Prevots DR. 2023. Environmental risk of nontuberculous mycobacterial infection: strategies for advancing methodology. Tuberculosis (Edinb) 139:102305. doi:10.1016/j.tube.2023.102305 36706504 PMC10023322

[57] Barnard E, Li H. 2017. Shaping of cutaneous function by encounters with commensals. J Physiol 595:437–450. doi:10.1113/JP271638 26988937 PMC5233660

[58] Salter SJ, Cox MJ, Turek EM, Calus ST, Cookson WO, Moffatt MF, Turner P, Parkhill J, Loman NJ, Walker AW. 2014. Reagent and laboratory contamination can critically impact sequence-based microbiome analyses. BMC Biol 12:87. doi:10.1186/s12915-014-0087-z 25387460 PMC4228153

[59] Corretto E, Antonielli L, Sessitsch A, Höfer C, Puschenreiter M, Widhalm S, Swarnalakshmi K, Brader G. 2020. Comparative genomics of Microbacterium species to reveal diversity, potential for secondary metabolites and heavy metal resistance. Front Microbiol 11:1869. doi:10.3389/fmicb.2020.01869 32903828 PMC7438953

[60] Han SK, Nedashkovskaya OI, Mikhailov VV, Kim SB, Bae KS. 2003. Salinibacterium amurskyense gen. nov., sp. nov., a novel genus of the family Microbacteriaceae from the marine environment. Int J Syst Evol Microbiol 53:2061–2066. doi:10.1099/ijs.0.02627-0 14657146

[61] Dahal RH, Kim J. 2019. Glaciihabitans arcticus sp. nov., a psychrotolerant bacterium isolated from Arctic soil. Int J Syst Evol Microbiol 69:2492–2497. doi:10.1099/ijsem.0.003520 31210627

[62] Kim J-J, Alkawally M, Brady AL, Rijpstra WIC, Sinninghe Damsté JS, Dunfield PF. 2013. Chryseolinea serpens gen. nov., sp. nov., a member of the phylum Bacteroidetes isolated from soil. Int J Syst Evol Microbiol 63:654–660. doi:10.1099/ijs.0.039404-0 22544793

[63] Li F, Hao X, Lu Q, Tuo L, Liu S, Zheng H, Sibero MT, Shen C, Sun C. 2023. Protaetiibacter mangrovi sp. nov., isolated from mangrove soil. J Antibiot 76:532–539. doi:10.1038/s41429-023-00627-w 37208458

[64] Wang J-J, Chen Q, Li Y-Z. 2018. Chryseolinea flava sp. nov., a new species of Chryseolinea isolated from soil. Int J Syst Evol Microbiol 68:3518–3522. doi:10.1099/ijsem.0.003022 30222097

[65] Heo J, Cho H, Kim MA, Hamada M, Tamura T, Saitou S, Kim S-J, Kwon S-W. 2019. Protaetiibacter intestinalis gen. nov., of the family Microbacteriaceae, isolated from gut of Protaetia brevitarsis seulensis, reclassification of Lysinimonas kribbensis Jang et al. 2013 as Pseudolysinimonas kribbensis gen. nov., comb. nov. and emended description of the genus Lysinimonas Jang et al. 2013. Int J Syst Evol Microbiol 69:2101–2107. doi:10.1099/ijsem.0.003444 31099733

[66] Lee SA, Heo J, Kim MA, Tamura T, Saitou S, Kwon S-W, Weon H-Y. 2021. Protaetiibacter larvae sp. nov. and Agromyces intestinalis sp. nov., isolated from the gut of larvae of Protaetia brevitarsis seulensis, reclassification of Lysinimonas yzui as Pseudolysinimonas yzui comb. nov. and emended description of the genus Pseudolysinimonas. Int J Syst Evol Microbiol 71. doi:10.1099/ijsem.0.004669 33913805

[67] Davis MJ, Gillaspie AG, Vidaver AK, Harris RW. 1984. Clavibacter: a new genus containing some phytopathogenic coryneform bacteria, including Clavibacter xyli subsp. xyli sp. nov., subsp. nov. and Clavibacter xyli subsp. cynodontis subsp. nov., pathogens that cause ratoon stunting disease of sugarcane and bermudagrass stunting disease. Int J Syst Bacteriol 34:107–117. doi:10.1099/00207713-34-2-107

[68] Jani K, Kajale S, Shetye M, Palkar S, Sharma A. 2021. Marisediminicola senii sp. nov. isolated from Queen Maud Land, Antarctica. Int J Syst Evol Microbiol 71. doi:10.1099/ijsem.0.004641 33439118

[69] Li A-H, Liu H-C, Xin Y-H, Kim S-G, Zhou Y-G. 2014. Glaciihabitans tibetensis gen. nov., sp. nov., a psychrotolerant bacterium of the family Microbacteriaceae, isolated from glacier ice water. Int J Syst Evol Microbiol 64:579–587. doi:10.1099/ijs.0.052670-0 24158943

[70] Li H-R, Yu Y, Luo W, Zeng Y-X. 2010. Marisediminicola antarctica gen. nov., sp. nov., an actinobacterium isolated from the Antarctic. Int J Syst Evol Microbiol 60:2535–2539. doi:10.1099/ijs.0.018754-0 19965991

[71] Altimira F, Yáñez C, Bravo G, González M, Rojas LA, Seeger M. 2012. Characterization of copper-resistant bacteria and bacterial communities from copper-polluted agricultural soils of central Chile. BMC Microbiol 12:193. doi:10.1186/1471-2180-12-193 22950448 PMC3496636

[72] Touceda-González M, Brader G, Antonielli L, Ravindran VB, Waldner G, Friesl-Hanl W, Corretto E, Campisano A, Pancher M, Sessitsch A. 2015. Combined amendment of immobilizers and the plant growth-promoting strain Burkholderia phytofirmans PsJN favours plant growth and reduces heavy metal uptake. Soil Biol Biochem 91:140–150. doi:10.1016/j.soilbio.2015.08.038

[73] Waigi MG, Kang F, Goikavi C, Ling W, Gao Y. 2015. Phenanthrene biodegradation by sphingomonads and its application in the contaminated soils and sediments: a review. Int Biodeterior Biodegradation 104:333–349. doi:10.1016/j.ibiod.2015.06.008

[74] Yabuuchi E, Kosako Y, Fujiwara N, Naka T, Matsunaga I, Ogura H, Kobayashi K. 2002. Emendation of the genus Sphingomonas Yabuuchi et al. 1990 and junior objective synonymy of the species of three genera, Sphingobium, Novosphingobium and Sphingopyxis, in conjunction with Blastomonas ursincola. Int J Syst Evol Microbiol 52:1485–1496. doi:10.1099/00207713-52-5-1485 12361250

[75] Tang S-K, Wang Y, Guan T-W, Lee J-C, Kim C-J, Li W-J. 2010. Amycolatopsis halophila sp. nov., a halophilic actinomycete isolated from a salt lake. Int J Syst Evol Microbiol 60:1073–1078. doi:10.1099/ijs.0.012427-0 19666809

[76] Dávila Costa JS, Amoroso MJ. 2014. Current biotechnological applications of the genus Amycolatopsis. World J Microbiol Biotechnol 30:1919–1926. doi:10.1007/s11274-014-1622-3 24557749

[77] Kisil OV, Efimenko TA, Efremenkova OV. 2021. Looking back to Amycolatopsis: history of the antibiotic discovery and future prospects. Antibiotics (Basel) 10:1254. doi:10.3390/antibiotics10101254 34680834 PMC8532670

[78] Song Z, Xu T, Wang J, Hou Y, Liu C, Liu S, Wu S. 2021. Secondary metabolites of the genus Amycolatopsis: structures, bioactivities and biosynthesis. Molecules 26:1884. doi:10.3390/molecules26071884 33810439 PMC8037709

[79] Satola B, Wübbeler JH, Steinbüchel A. 2013. Metabolic characteristics of the species Variovorax paradoxus. Appl Microbiol Biotechnol 97:541–560. doi:10.1007/s00253-012-4585-z 23192768

[80] Hirsch AM, Valdés M. 2010. Micromonospora: an important microbe for biomedicine and potentially for biocontrol and biofuels. Soil Biol Biochem 42:536–542. doi:10.1016/j.soilbio.2009.11.023

[81] Yang G, Han L, Wen J, Zhou S. 2013. Pseudomonas guangdongensis sp. nov., isolated from an electroactive biofilm, and emended description of the genus Pseudomonas Migula 1894. Int J Syst Evol Microbiol 63:4599–4605. doi:10.1099/ijs.0.054676-0 23918787

[82] Monciardini P, Cavaletti L, Schumann P, Rohde M, Donadio S. 2003. Conexibacter woesei gen. nov., sp. nov., a novel representative of a deep evolutionary line of descent within the class Actinobacteria. Int J Syst Evol Microbiol 53:569–576. doi:10.1099/ijs.0.02400-0 12710628

[83] Makkar NS, Casida LE. 1987. Cupriavidus necator gen. nov., sp. nov.; a nonobligate bacterial predator of bacteria in soil. Int J Syst Bacteriol 37:323–326. doi:10.1099/00207713-37-4-323

[84] Vandamme P, Coenye T. 2004. Taxonomy of the genus Cupriavidus: a tale of lost and found. Int J Syst Evol Microbiol 54:2285–2289. doi:10.1099/ijs.0.63247-0 15545472

[85] BacDive. 2024. The bacterial diversity metadatabase. Available from: https://bacdive.dsmz.de/. Retrieved 12 Sep 2024.

[86] Ķimsis J, Pokšāne A, Kazarina A, Vilcāne A, Petersone-Gordina E, Zayakin P, Gerhards G, Ranka R. 2023. Tracing microbial communities associated with archaeological human samples in Latvia, 7-11th centuries AD. Environ Microbiol Rep 15:383–391. doi:10.1111/1758-2229.13157 37057308 PMC10472514

