# Supplementary material for: Characterization of ancient DNA preservation in copper-patinated human bone and tooth samples from Latvia
Source: Microbiol Spectr. 2025 Aug 12;13(9):e02705-24. doi: 10.1128/spectrum.02705-24 (PMC12403904; doi:10.1128/spectrum.02705-24)
Supplement: Supplemental figures — Figures S1 to S6. [file spectrum.02705-24-s0001.pdf]

Supplementary Figure 1

Location of Lejasbitēni burial site in Latvia, which is the origin site for all archaeological samples used in this study.

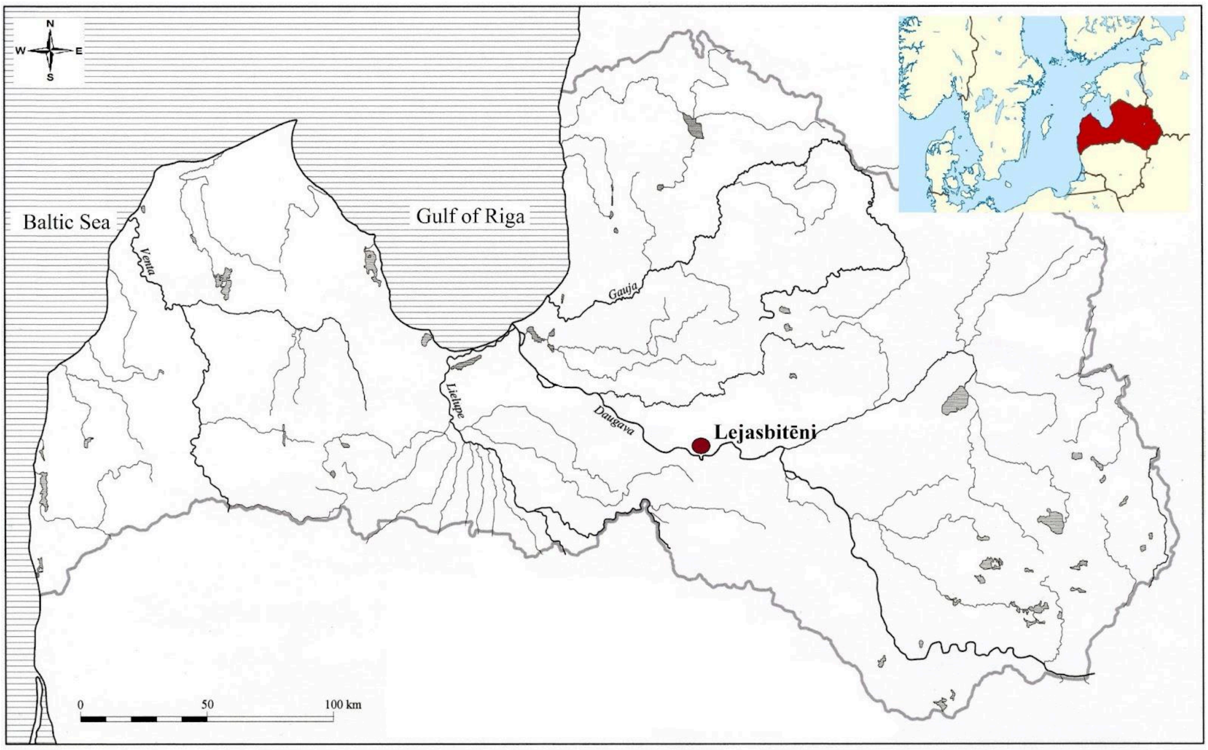

Red dot: Location of Lejasbitēni burial site in Latvia, which is the origin site for all samples used in this study.

Lejasbitēni is a 7th - 10th century CE burial site located on the right bank of Daugava river. Currently the site is under water due to artificial flooding because of a nearby Pļaviņu hydroelectric station.

Supplementary Figure 2
mapDamage plots of sequencing reads aligning to human genome reference for negative control samples used in this study. None of the negative controls show an authentic aDNA damage pattern.

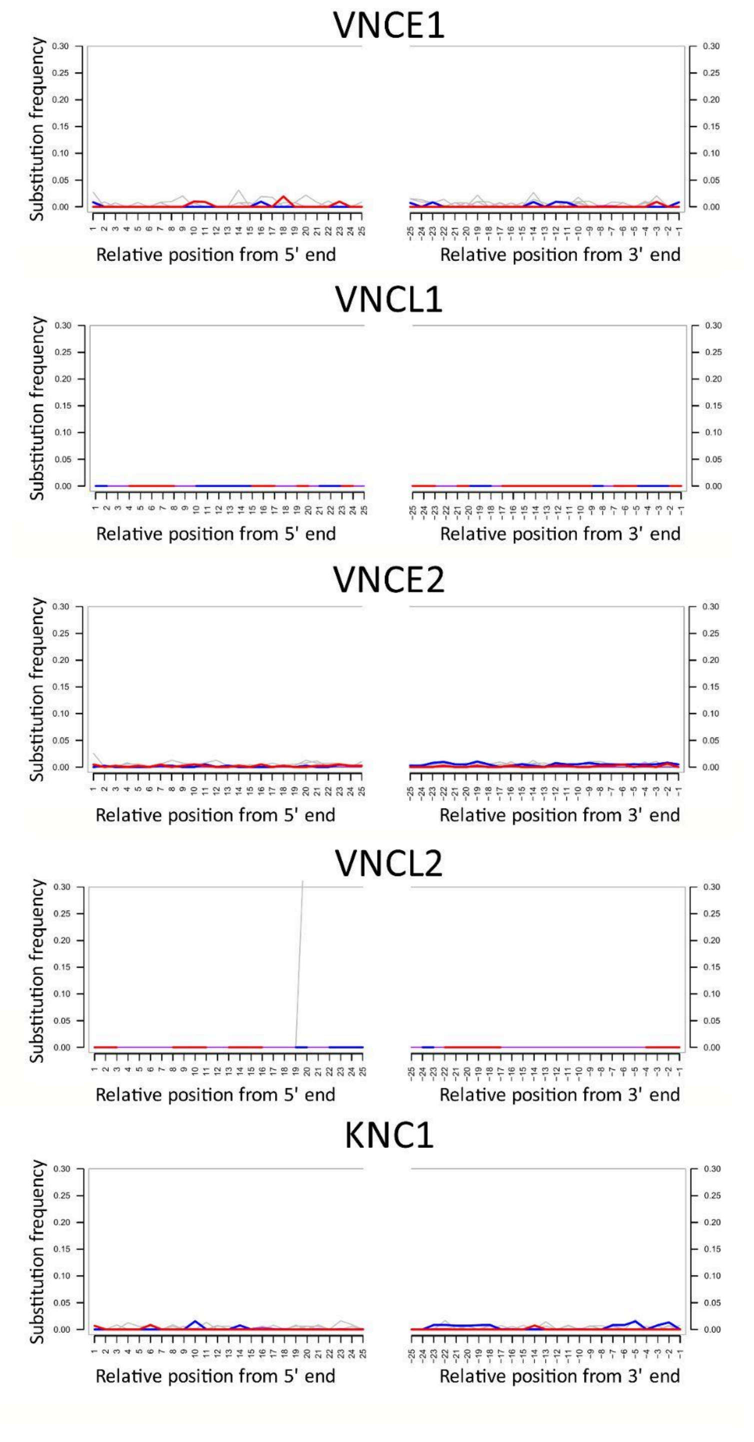

Assigned colors: red – C to T substitutions; blue – G to A substitutions; grey – all other substitutions. Orange – soft-clipped bases; green – deletions relative to the reference; purple – insertions relative to the reference.

Supplementary Figure 3

mapDamage plots of sequencing reads aligning to human genome reference. DNA was extracted from tooth samples using Protocol V. Graph shows increased proportion of G to A substitutions at the 3’ ends for unpatinated samples VNPT1 and VNPT2, which is characteristic of aDNA damage. Due to the specific polymerase included in the library construction kit used for this study no C to T substitutions at 5’ ends could be observed. Samples with patination do not show such a signal.

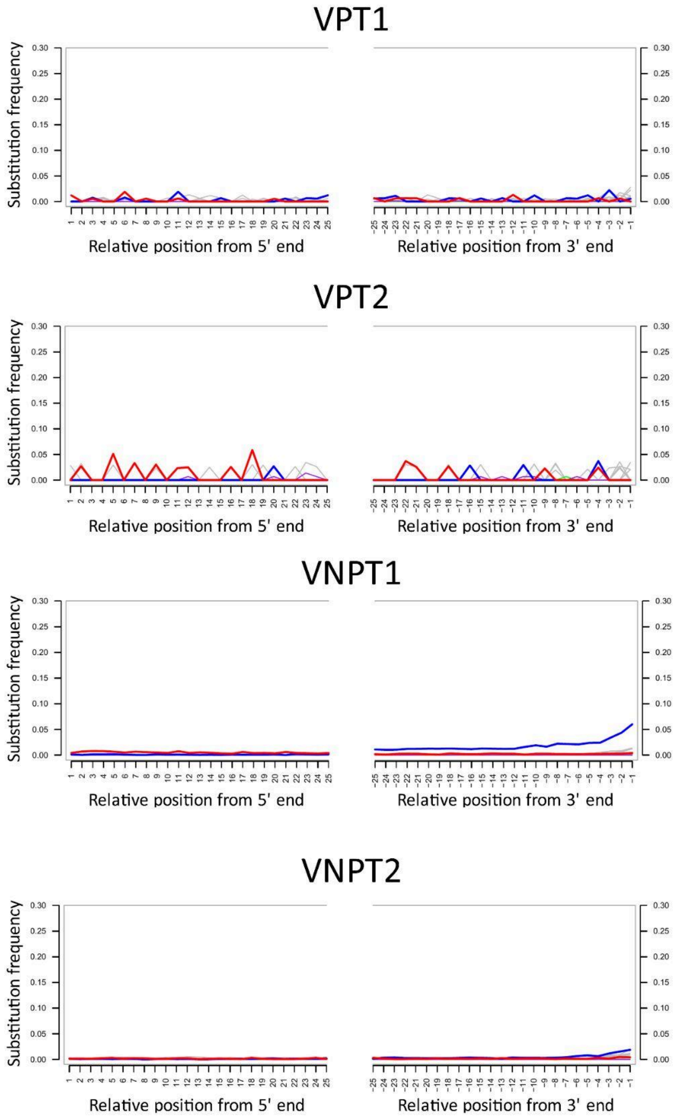

Assigned colors: red – C to T substitutions; blue – G to A substitutions; grey – all other substitutions. Orange – soft-clipped bases; green – deletions relative to the reference; purple – insertions relative to the reference.

Supplementary Figure 4

mapDamage plots of reads aligning to human genome reference. DNA was extracted from bone samples using Protocol V. Graph shows increased proportion of G to A substitutions at the 3' ends for unpatinated samples (VNPB1 and VNPB2), which is characteristic of aDNA damage. Due to the specific polymerase included in the library construction kit used for this study no C to T substitutions at 5' ends could be observed. Sample with patination (VPB1) shows a very weak damage pattern.

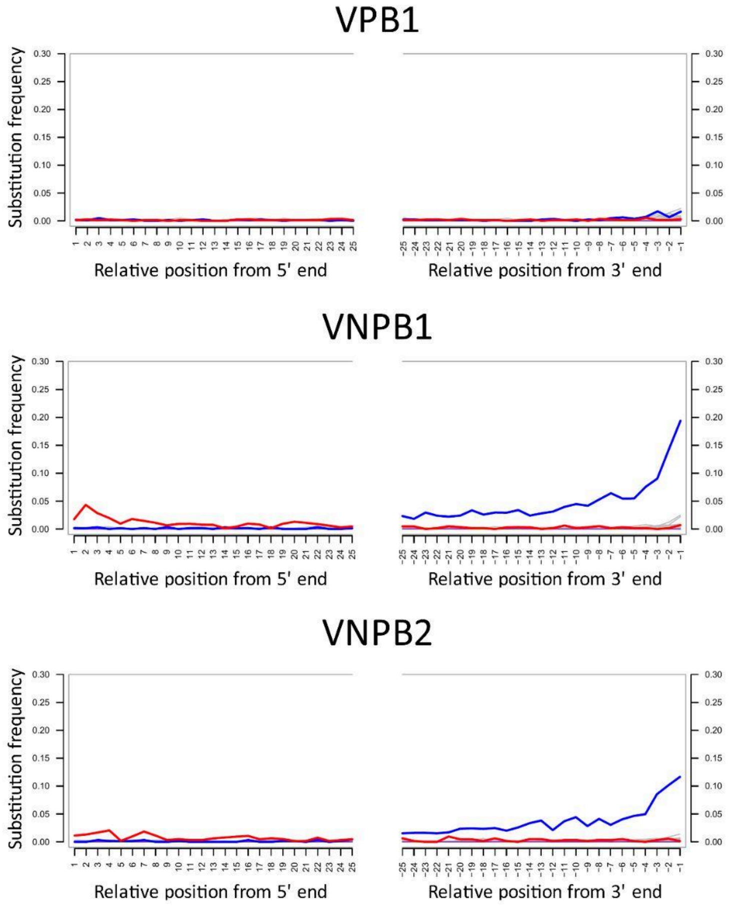

Assigned colors: red – C to T substitutions; blue – G to A substitutions; grey – all other substitutions. Orange – soft-clipped bases; green – deletions relative to the reference; purple – insertions relative to the reference.

Supplementary Figure 5

mapDamage plots of reads aligning to human genome reference. DNA was extracted from tooth samples using Protocol K. Graph shows increased proportion of G to A substitutions at the 3’ ends for unpatinated sample (KNPT1), which is characteristic of aDNA damage. Due to the specific polymerase included in the library construction kit used for this study no C to T substitutions at 5’ ends could be observed. Sample with partial patination (KPT1) exhibits a damage pattern while severely patinated sample (KPT2) does not.

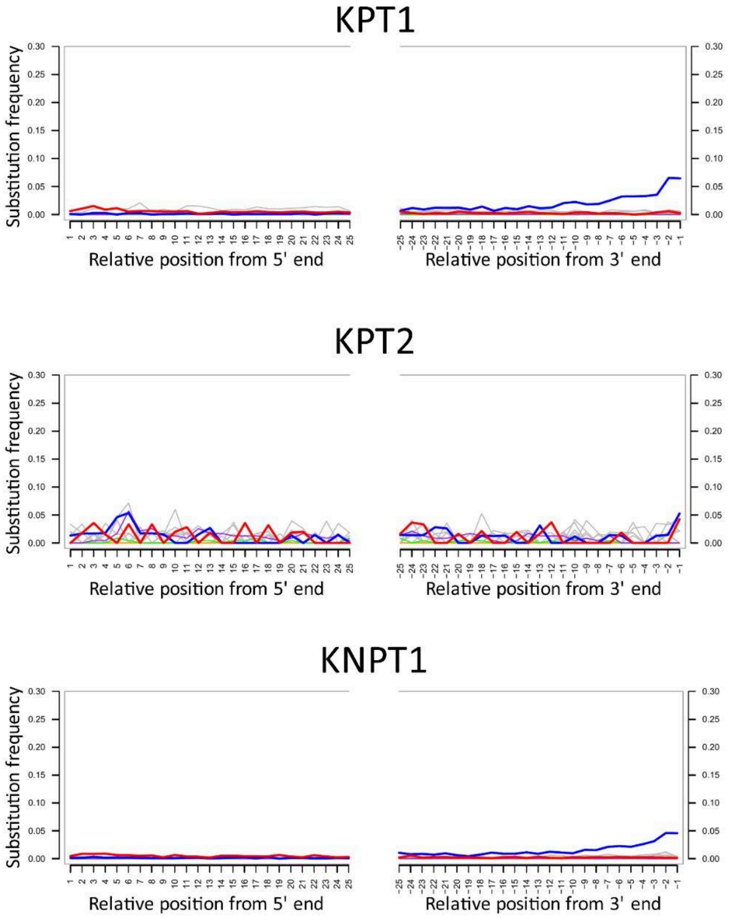

Assigned colors: red – C to T substitutions; blue – G to A substitutions; grey – all other substitutions. Orange – soft-clipped bases; green – deletions relative to the reference; purple – insertions relative to the reference.

Supplementary Figure 6

Relative microbial genera abundance plot for negative control samples.

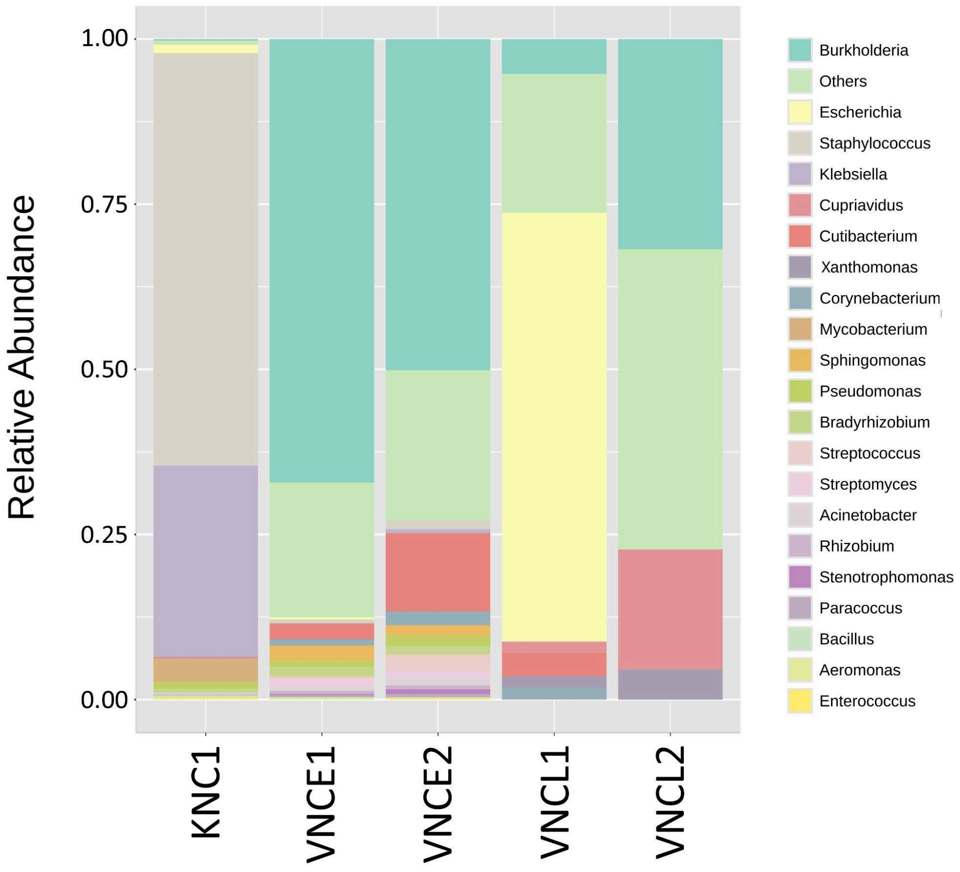

Relative abundance plot shows difference between Protocol K and Protocol V negative controls. Sequencing library construction negative controls (VNCL1, VNCL2) show lesser complexity than DNA extraction negative controls (KNC1, VNCE1 and VNCE2).
